# Best practices for government agencies to publish data: lessons from COVID-19

**DOI:** 10.1016/S2468-2667(24)00073-2

**Published:** 2024-05-01

**Authors:** Bastian Herre, Lucas Rodés-Guirao, Edouard Mathieu, Hannah Ritchie, Charlie Giattino, Joe Hasell, Saloni Dattani, Esteban Ortiz-Ospina, Max Roser

**Affiliations:** a Our World in Data, Oxford, UK; b Oxford Martin Programme on Global Development, University of Oxford, Oxford, UK; c Department of Social Policy and Intervention, University of Oxford, Oxford, UK

## Abstract

Without data, knowing how to respond to the COVID-19 pandemic would have been impossible. Data were crucial to understanding how the disease spread and which efforts successfully protected people. Yet, national agencies often did not publish their data in an optimal way, which made responding to the pandemic challenging. Therefore, learning from what went well and what did not for the future is crucial. Drawing on our first-hand experience of republishing COVID-19 data, we identify seven best practices for how to publish data in an optimal way: collect the data that are relevant; make them comparable; clearly document the data; share them frequently and promptly; publish data at a stable location; choose a reusable format; and license others to reuse the data. These best practices are straightforward, inexpensive, and achievable, with some countries already having implemented most of them during the COVID-19 pandemic. More government agencies following these best practices will enable others to access their data and address the world's public health challenges—including the next pandemic.

## Introduction

During the COVID-19 pandemic, data were crucial to decide how to react to the spread of the virus: data on cases revealed who was at risk from the disease; data on hospitalisations, deaths, and excess mortality provided information on the severe impacts on people's health; and data on policy responses, medical treatments, and vaccinations showed which efforts successfully protected people.

Yet, national agencies often did not publish their data in an optimal way, which made responding to the pandemic challenging. Others—teams of university researchers, data journalists, and our team at Our World in Data—republished the data in an effort to make the data easier to access and understand. In this Viewpoint, we summarise what we learnt from this work and how government agencies could have been more successful in making their data available.

We at Our World in Data have first-hand experience of republishing COVID-19 data, as we built and maintained a widely used global database about the pandemic. The database assembled relevant metrics—such as on testing, hospitalisations, and vaccinations—which we compiled from countries worldwide and then harmonised and republished.^1^ The database was relied upon by many international organisations, including WHO, international media, and governments in many countries. Our data have been viewed billions of times by people worldwide.

Drawing on this experience, we have identified best practices for public agencies to publish their data, to enable others—their colleagues in government institutions, their citizens, the media, researchers, and data providers like us—to access and understand the data. Agencies should: (1) collect the data that are relevant; (2) make the data comparable; (3) clearly document the data; (4) share the data frequently and promptly; (5) publish the data at a stable location; (6) choose a reusable data format; and (7) license others to reuse the data. These best practices complement others, such as the findability, accessibility, interoperability, and reusability principles,^2^ and build on the work of others^3^ and our own^4^, ^5^ during the pandemic.

## Data relevance

Collecting the data that are relevant means focusing on the crucial metrics to answer the questions that decision makers and the public have. The data should not leave out anything essential or include anything that would divert resources and attention away from the most relevant metrics. The most relevant data during the COVID-19 pandemic included data on cases, hospitalisations, deaths, and excess mortality to know how fast the disease was spreading and how severe it was; data on testing to know how well the spread of the disease was monitored; and data on hospital occupancy, policy responses, and vaccinations to know how supported people infected were and how protected healthy people were.

A major example of not collecting the relevant data during the early stages of the pandemic was the lack of testing data in several countries. This insufficiency meant that people often did not know whether their country was still largely unaffected by the pandemic, or whether case counts were only low because few people were being tested. During later stages of the pandemic, governments could have used the increased testing capacities better: for instance, they could have regularly tested representative samples of the population. Alternatively, a cheaper and helpful alternative was to publish data on the share of positive tests to learn more about the under-reporting of cases and deaths.^6^

Another example of not collecting the relevant data is health outcomes—hospitalisations and deaths—by vaccination status. Even today, these data are not available for the majority of countries. Understanding who gets hospitalised and dies from COVID-19 is crucial information for deciding whether to get vaccinated. Research showed that people cared about vaccines' efficacy.^7^ In some countries, governments might have been held back by the absence of a vaccination database that told health-care providers whether a patient had been vaccinated or not. Even in the absence of such a database, these data could have been collected by asking the patients or their relatives.

Some agencies succeeded in collecting much or all of the relevant data. Chile's Ministry of Health, for instance, opened a dedicated GitHub repository to publish close to 100 datasets, including deaths from COVID-19 by whether and how often people have been vaccinated.^8^ Many governments did better over time. The Pacific Community, for example, added data on vaccination boosters after we inquired. Sometimes, we were able to improve on the work of public agencies by leveraging the data they shared to calculate more relevant metrics. One such instance was that we calculated the share of positive tests.

The data that are relevant will be different for other areas and might even be different for another public health crisis. International health agencies could play a crucial part in identifying the important metrics and recommending which data should be collected. National agencies can investigate which data could answer the pressing questions of their governments, and then prioritise the collection of the corresponding data.

## Data comparability

Making the data comparable means prioritising that the data can be compared across time and geography. Ideally, comparability is achieved within and across countries by using the same definitions and methods to collect data. Comparable data matter because it makes identifying inequities, learning from others, and reproducing the best policies possible.

An example of not making the data comparable during the pandemic was cross-national testing. Comparisons between countries were difficult as some countries counted the number of tests done whereas others counted the number of people tested. This discrepancy made ascertaining whether a country's cases and deaths were lower than another country more difficult because the difference could have been because of less testing or because the policies were working better.

Another example comes from vaccination data, in which some countries reported people as vaccinated when they had received at least one dose, whereas others only reported people who had received all doses required by the manufacturer as vaccinated. This difference in definition made determining which vaccination campaigns were working best across all countries difficult.

Such differing definitions meant that comparisons between countries were often inexact or that we had to recalculate figures to make them comparable and show statistics different from the official ones. Alternatively, we shared similar but distinct metrics separately to at least allow for partial comparisons among countries that collected one of the metrics. Some governments recognised the problem of non-comparable data and changed what they shared. For instance, the Austrian Government asked us how they can make their data comparable to those of other countries.

Looking ahead, international health agencies have an especially important part to play. They are in the best position to give recommendations for how to measure the relevant metrics to ensure that comparable data are collected, and then to assemble the country data into a cross-national dataset.

## Data documentation

Clearly documenting the data primarily means that the data providers describe what exactly their data are measuring. Additionally, data providers also documenting what time and geography the data cover, how they collected the data, and how they addressed any challenges is helpful. In the best cases, providers also justify their procedures, acknowledge any remaining shortcomings, share the raw data and the code of how they processed it (eg, calculating one indicator on the basis of several others), and respond to user questions via email and GitHub.

Good documentation should be unambiguous and easy to understand; it includes good structuring, avoiding technical terms, and having accompanying simplified descriptions if appropriate. Documenting the data well matters as it allows experts and laypeople alike to understand the data, evaluate its strengths and weaknesses, and identify what questions can and cannot be answered with the data.

Many countries did not document their data well during the pandemic. Some agencies did not provide any descriptions of their testing and vaccination data, often because the agencies shared the data only on social media, where they had little room to describe it. This approach meant we and other users had to ask the agencies and engage in a back-and-forth to understand the data. We then created our own detailed documentation and made the data and documentation publicly available.

Another recurring problem was that data providers did not make clear that they were only reporting confirmed deaths from COVID-19. Media outlets often misunderstood these data and published articles or data visualisations that suggested that countries with the highest number of confirmed deaths were those affected most, ignoring the large differences in testing between countries. In our work, we emphasised that testing differences were crucial, that we were only reporting confirmed cases and deaths, and that these are often only a fraction of the total number of COVID-19 cases and deaths. Some agencies documented their data well. One example was Malaysia's Ministry of Health, which published clear descriptions of each variable, shared how they processed the data, and how the data could be used.^9^

## Data sharing frequency

Sharing data frequently and promptly means that the data are shared at the frequency that is needed—daily data for the rapidly developing pandemic, at least for the most important metrics. Some countries failed to do this during the pandemic, and only reported data infrequently. Other countries did well later during the pandemic, including Argentina and South Korea, who released new vaccination data daily.

## Data publication location

Data should be published at a stable location, meaning on the same website and under an identical and accessible uniform resource locator (URL), with the new information being added to the existing dataset. The URL should not change with the addition of new data and should be accessible from anywhere in the world, and new data should not overwrite the old. A stable location matters because people then will have an easy time finding the data again and can automate retrieving data for reuse and analysis.

Many countries did not publish data at a stable location during the pandemic. For example, numerous countries relied on social media posts or videos to share the latest data with the public. Researchers, journalists, and data aggregators had to check these social media accounts several times a day, find the relevant post and video among others, and read and watch each of them to find the latest data.

Other countries did better and published the data on the same website, but frequently changed the URL. Whenever the URL was changed, we had to find the new URL and adapt our code to extract the data. There were, however, positive examples: Italy's Government shared data on GitHub from early in the pandemic;^10^ and the Government of the Dominican Republic reached out to us to learn how to make their data easier and more reliable to access.

## Data format

The best practice is to publish the data in a format that a computer can automatically process. Examples of machine-readable formats are comma-separated values (CSVs) and JavaScript Object Notation. The best specific format depends on the structure of the data, such as tabular or geographical data. Ideally, the metadata (variable descriptions, units, etc) are also shared in a machine-readable format, and the data follow common standards, such as ISO 8601 for writing dates, Snake case for writing variable names, and UTF-8 for encoding (as much as possible given different national languages).

Reusable data formats matter because the data that agencies put out are often the input for the work of others, whose exploration and use of the data only starts there. A dashboard accompanying the data can allow people to explore it easily, but it is no substitute for a machine-readable spreadsheet that allows many other uses.

Countries often fell short of these standards during the pandemic. Some agencies shared testing and vaccination data through screenshots of spreadsheets. Many used PDF files or Hypertext Markup Language (HTML) tables, which are not much easier to reuse. PDF files require transcription of the data by hand or optical character recognition, and HTML tables require writing and frequently patching additional code to access them.

We reshared these data in a reusable format. We also worked to support public agencies directly by distributing a template on how to best format their vaccination data. Some agencies did well, and chose reusable data formats and common standards, such as Sciensano, the Belgian Institute for Health.^11^

## Data licensing and reuse

Licensing data for reuse means the data come with a license that allows other people to reuse them, such as a permissive Creative Commons Attribution license. Those accessing the data must also be able to see the license. The license should be shown close to where the data are displayed or can be downloaded.

Licensing matters because people will have many questions about the data that can only be answered by further analysing them. If they do not have the license to do so, these questions will remain unanswered and decision makers and the public will be less informed.

Many countries during the pandemic did not explicitly license others to reuse their data. Because the stakes for public health were high and the data were published by governments, we and many others operated under the assumption that we were licensed to use the data. No government ever took issue with data reuse. But we take licensing seriously and are concerned. Clarity would have been beneficial—and might have led to more people using the data.

## Conclusion

A lot of the data published by government agencies during the pandemic did not follow these best practices. Often, others—such as teams of university researchers, data journalists, and our team at Our World in Data—had to improve available data to make them easier to access and understand. Sometimes even individuals became key people in making data accessible and understandable, such as in Peru^12^ and Uruguay.^13^ Some countries, such as Italy and Malaysia, already came close to all the best practices during the pandemic, and many others followed at least some of the practices or improved over the pandemic.

Following best practices for publishing data is feasible and is often easy. Some recommendations—such as providing all relevant data—will go beyond what some poorly funded agencies can provide, but most recommendations—such as publishing the data in a CSV format at a stable location—are straightforward and cheap. When we shared these recommendations with data publishers during the pandemic, they often implemented them, which suggests that public agencies do not have objections against these best practices, but that they simply did not consider them.

If government agencies adopt these seven best practices, everyone will be in a better position to understand the world's most important public health issues and how to address them—including the next pandemic.

## Declaration of interests

We declare no competing interests.
