# Correction to *Lancet Public Health* 2024; 9: e407–10

**DOI:** 10.1016/S2468-2667(24)00121-X

**Published:** 2024-05-31

*Herre B, Rodés-Guirao L, Mathieu E, et al. Best practices for government agencies to publish data: lessons from COVID-19.* Lancet Public Health *2024; **9:** e407–10*—In this Viewpoint, the second sentence of the fourth paragraph of the Data relevance section should have read “Chile's Ministry of Health, for instance, collected a large number of useful metrics, including deaths from COVID-19 by whether and how often people have been vaccinated, which the Ministry of Science then published in a dedicated GitHub repository”. These correction has been made as of May 30, 2024.

